# Presupposition processing declines with age

**DOI:** 10.1007/s10339-022-01088-z

**Published:** 2022-04-20

**Authors:** Robert Reinecke, Simona di Paola, Filippo Domaneschi, Marion Fossard

**Affiliations:** 1 Institut des Sciences Logopédiques, University of Neuchâtel, Neuchâtel, Switzerland; 2 FORS – Swiss Centre of Expertise in the Social Sciences – Bâtiment Géopolis, 5th Floor Reception Desk, 1050 Lausanne, Switzerland; 3 Laboratory of Language and Social Cognition, Corso Podestà, 2, Room 3C3, 16128 Genova, Italy; 4 DAFIST, Laboratory of Language and Social Cognition, Via Balbi 30, 16128 Genova, Italy

**Keywords:** Language processing, Experimental pragmatics, Cognitive load, Aging, Definite descriptions, Change-of-state verbs

## Abstract

The present study investigates the processing of presuppositions across the life span and extends the findings of the only available study on presupposition processing and typical aging by Domaneschi and Di Paola (J Pragmat 140:70–87, 2019). In an online and offline task, we investigate the impact of cognitive load during the processing and recovery of two presupposition triggers—definite descriptions and change-of-state verbs—comparing a group of younger adults with a group of older adults. The collected experimental data show that (1) presupposition recovery declines during normal aging, (2) presupposition recovery of change-of-state verbs is more cognitively demanding for older adults than the recovery of definite descriptions, and lastly (3) presupposition recovery for the change-of-state verb *begin* is more demanding than the change-of-state verb *stop*. As of today, few works have directly investigated presupposition processing across the life span. To the best of our knowledge, this is the first work revealing that cognitive load directly impacts the recovery of presuppositions across the life span, which in turn suggests an involvement of verbal working memory.

## Introduction

### Presuppositions

Presupposition is background information that is taken for granted (Stalnaker 1974). For example, the utterances.

1. John has stopped smoking.
2. The painting was stolen.

presuppose (1a) and (2a) below:

- John used to smoke.
- There was a painting.

Presuppositions are carried by *presupposition triggers*, namely lexical items and syntactic constructions that activate a presupposition. Instances of presupposition triggers are iterative expressions, factive verbs, focus-sensitive particles or, as illustrated, respectively, in (1) and (2), change-of-state verbs such as *to stop*, and definite descriptions such as *the painting* in (2) (e.g., Karttunen 1974; Levinson 1983).

When a speaker utters sentences such as (1) or (2), three possible outcomes may occur. First, if the presupposition is entailed by the context before the utterance time, then it is said to be *satisfied* and the context can be updated with the assertive component of the utterance. Conversely, if the presupposition is not part of the common ground, this leads to *presupposition failure*. In such a case, the hearers can react in two different ways. One possibility is that if the presupposition is controversial or surprising, then the hearers can reject an utterance like (1) as inappropriate by replying ‘Hey, wait a minute! I had no idea that John used to smoke?’ (von Fintel 2004). Alternatively, failure can be repaired to make sense of the utterance felicity. The mechanism underlying failure repair is *accommodation* (Lewis 1979; Heim 1982), i.e., the process whereby a presupposition that is not satisfied by the context is introduced in the context set to allow for the contextual update.^1^

The theoretical literature on presupposition generally agrees on the intuition that not all classes of presupposition triggers behave equally in the way they activate a presupposition.^2^ However, several theoretical distinctions have been proposed in this respect. For instance, Glanzberg (2003, 2005) distinguishes between *strong* and *weak presupposition triggers*. For the former, accommodating the corresponding presupposition in case of failure is supposed to be mandatory in order to preserve the utterance felicity. By contrast, for the latter, accommodating the presupposition to repair the failure is optional because even without the presupposed content the utterance still makes sense, despite being partially infelicitous. Within this distinction, both change-of-state verbs and definite descriptions are classified as strong presupposition triggers: the presupposed content carried by these triggers provides a meaningful contribution to the propositional content of the utterance, therefore, repairing the context in case of failure becomes necessary to understand the utterance (e.g., ‘John has stopped smoking’ is true only if John smoked; similarly, ‘The painting was stolen’ is true only if there was a painting). By contrast, instances of weak presupposition triggers would be focus-sensitive particles such as *too* or iterative expressions such as *again*: here, the failure would induce an optional repair because the context can still be meaningfully updated with the assertive component of the utterance (e.g., ‘John smokes too’ or ‘A painting was stolen again’). Beyond Glanzberg, Zeevat (1992) distinguishes between *resolution* and *lexical* triggers: resolution triggers, such as definite descriptions, would involve the anaphoric retrieval of an entity or event in the common ground; lexical triggers, such as change-of-state verbs, would activate no anaphoric retrieval since their conventional meaning already encodes a precondition for their asserted content (e.g., John would have not stopped smoking if he did not smoke before).

### The cognitive costs of presupposition processing

A wealth of experimental research on presupposition investigated the cognitive cost of processing presupposition and which linguistic factors impact the cognitive demands, using both psycholinguistic (mostly reading and response times) and neurolinguistic (i.e., ERPs) paradigms.

Several behavioral experiments show not only that presuppositions are evaluated in online sentence comprehension (e.g., Schwarz 2007) and that presupposing utterances elicit longer reading times than non-presupposing utterances (e.g., Tiemann et al. 2011), but also that at least two main factors crucially modulate the cognitive cost of presupposition processing.^3^ First, the availability in context of the presupposed content. Accommodating a presupposition elicits greater cognitive costs than processing a presupposition that is satisfied by the context, both in online and offline processing and across a variety of triggers. For instance, definite descriptions require longer reading times when they are presented without a supportive context as compared to when context is provided (Haviland and Clark 1974; Yekovich and Walker 1978; Arnold et al. 2000)^4^. Additionally, the extra costs associated with accommodation have been replicated by evidence on other types of triggers. For example, accommodating a presupposition triggered by the German additive particle *auch* (‘too’) takes longer than processing a satisfied presupposition of *auch* in intra-sentential contexts (Schwarz 2007). A similar pattern emerged for the presupposition of *wieder* (‘again’) which, within a word-by-word reading time paradigm, elicited longer reading times at the critical region when accommodation was required as compared to satisfaction (Tiemann et al. 2015). Consistently, in a word-by-word reading time experiment, Domaneschi and Di Paola (2018) found greater cognitive costs for presupposition accommodation than satisfaction during both online (i.e., longer reading times at the critical regions) and offline (i.e., longer response times to verification questions) processing and across a variety of triggers that included definite descriptions and change-of-state verbs.^5^ Taken together, these results suggest higher cognitive costs for presupposition accommodation, independently of the specific type of trigger in use. This extra effort has been overall interpreted as reflecting the costs for context repair.

Second, the specific category of trigger also has an impact on the cognitive cost of presupposition processing. Domaneschi and Di Paola (2018) found processing differences between trigger types at different regions of the sentence using a word-by-word reading time paradigm. For instance, while definite descriptions elicited longer reading times at the word following the triggering point (i.e., the trigger itself, where the hearer is alerted that the context will have to entail a given proposition to make sense of the utterance), other triggers such as, for example, iterative expressions elicited longer reading times more forward in the sentence, at the computation point (i.e., where the content of the presupposition is actually processed). In addition, the results of their study also revealed processing differences across triggers when participants were asked to recover the presupposed information in an offline verification task. Based on Zeevat (1992), the authors attribute this pattern to the resolution versus lexical nature of the triggers under scrutiny: recovering the presupposition of a resolution trigger such as definite descriptions, that is inferred via the anaphoric search of a suitable antecedent, was costlier than recovering the presupposition of a lexical trigger such as change-of-state verbs, that is derived via direct logical implication.

The idea that different classes of triggers differently modulate the cognitive costs of presupposition processing has also been investigated with more fine-grained methods such as event-related potentials, and results corroborate the behavioral patterns. In fact, both definite descriptions and change-of-state verbs elicit N400 and P600 effects when processing requires accommodation as compared to when the context satisfies their presupposition (definite descriptions: Burkhardt 2006; Schumacher and Hung 2012; Wang and Schumacher 2013; definite descriptions and change-of-state verbs: Domaneschi et al. 2018). Importantly, this biphasic pattern seems modulated by the type of trigger: while definite descriptions, categorized as a referential anaphoric trigger in Zeevat’s (1992) proposal, elicit a more prominent N400, whereas change-of-state verbs, a lexical trigger according to Zeevat (1992) elicit a more prominent P600 (Domaneschi et al. 2018). The neural correlates have been interpreted along the lines of a Linking–Updating mechanism (Schumacher and Hung 2012). The more prominent N400 for definite descriptions would mirror the higher linking costs underlying the earlier search for a suitable antecedent. Differently, the more prominent P600 for change-of-state verbs would reflect the cost to update the mental model with the presupposed information.

Interestingly, it has been reported that the cognitive load of presupposition processing does not depend only on the availability in context of the presupposition and the specific class of trigger, but also on the complexity of the mental representation involved. Domaneschi et al. (2014) used a dual task to investigate (i) Glanzberg’s (2003) proposal that, in case of failure, the optional vs. mandatory processing of a presupposition depends on the category of the trigger at stake and (ii) differences in the cognitive demands to process the presupposition activated by different types of triggers. In their study, participants were asked to perform two tasks: first, they listened to a set of stories that contained several trigger types and, after each story, they responded to verification questions on the content of the presupposed information. The trigger types were definite descriptions, change-of-state verbs, factive verbs, iteratives, and focus-sensitive particles (e.g., respectively, *The X*, *to stop*, *to regret*, *again*, *too*) and were all presented within a context that did not satisfy their presuppositions. Second, while listening to the stories and answering to the questions, participants were asked to keep in mind either one (i.e., low cognitive load) or three (i.e., higher cognitive load) figures for later recall. In other words, the authors were interested in investigating whether the level of interference—low vs high—has an impact on presupposition recall. This study revealed interesting results. First, high overall accuracy scores (i.e., average of the low and high cognitive load condition) were obtained for factive verbs and definite descriptions, an intermediate score was obtained for change-of-state verbs and lower accuracy scores were reported for focus-sensitive particles and iteratives. Most interestingly, the interference effect emerged only with change-of-state verbs and iterative expressions: contrary to the other triggers, less correct answers were provided on the presuppositions of these triggers in the condition of low (accuracy scores for change-of state verbs and focus-sensitive particles: 83% and 65%, respectively) vs. high load (accuracy scores for change-of state verbs and focus-sensitive particles: 65% and 49%, respectively).^6^ The authors interpret these results as suggesting that both change-of-state verbs and iterative expressions are more cognitively demanding categories of triggers as compared to definite descriptions, factive verbs, and focus particles. The extra costs would be bound to the more complex nature of their mental representation; in fact, both trigger types imply a representation of temporally displaced events that includes the representation of an event at a previous time (e.g., X smokes) as well as the representation of an event at the time of the utterance (e.g., X has given up smoking). Based on this, the authors conclude that whether repairing the failure is mandatory or not does not primarily affect the cognitive load associated with a given presupposition trigger; rather, the complexity of the trigger underlying mental representation burdens presupposition processing.

To summarize, experimental research on presupposition overall suggests that presupposition processing comes at a cost. Crucially, this cost is not always the same; rather it depends on (1) whether or not a presupposition needs to be accommodated and (2) on which trigger is at stake. Last but not least, the complexity of the mental representation of certain classes of triggers influences the processing costs: processing the presuppositions of change-of-state verbs, whose mental representation involves temporally displaced events, seems costlier than processing the presuppositions of triggers with a less complex mental representation, such as definite descriptions.

### Presupposition processing in late adulthood: Domaneschi and Di Paola (2019)

Language abilities do not remain stable across the life span. Empirical research increasingly reports consistent patterns of variation from development to late adulthood and across all language sub-systems, from phonology and syntax to semantics and pragmatics (for a comprehensive up-to-date review, see for instance Kidd et al. 2018). Studying linguistic variation across the life span is twofold. First, life span is a proficient testing ground to investigate the cognitive and linguistic underpinnings of a given linguistic phenomenon; second, studying life span-related effects on a given linguistic ability informs us about the complex interplay between cognition and life span itself.

Surprisingly, research on presupposition processing across the life span is still very limited. A few studies investigated children’s presuppositional skills and these overall reported developmental patterns (e.g., Hüttner et al. 2004; Bergsma 2006; Höhle et al. 2009; Berger and Höhle 2012; Dudley et al. 2015; see Pouscoulous 2013 for a review). Even more surprisingly, despite the extensive literature on pragmatic abilities in normal aging (see Messer 2015), almost nothing is known about presuppositional skills in late adulthood.

Before turning to the only study that explicitly investigated presuppositional skills in late adulthood, one of the first experiments investigating inference processing across the life span will be presented. Zacks et al. (1987) investigated inference processing across the life span. In their experiment, young adults (mean age = 20.4 years) and older adults (mean age = 73.2 years) were asked to respond to verification questions after listening to a story that could contain either explicit, expected, or unexpected information. In the explicit condition, the information was explicitly provided, whereas only strong contextual cues were given in the expected condition and misleading cues were given in the unexpected condition. Their results indicate that accuracy scores between the young and older adults do not differ in the explicit condition; however, significant age differences arise in the expected and unexpected condition (accuracy difference: 17.7% and 15.1%, respectively). Moreover, older adults’ performance gradually decreases with increasing complexity (explicit accuracy > expected accuracy > unexpected accuracy, respectively, 85.4%, 72.4%, and 65.6%). In summary, this study provides first evidence that inference making deteriorates across the life span.

The previously presented findings seem relevant for the investigation of presupposition processing across the life span. In fact, presuppositions convey information that are communicated implicitly. Moreover, during the process of accommodation, the presupposition is not yet mutually agreed upon, but must be tacitly accepted in order to become part of the common ground. To the best of our knowledge, the only study on presuppositional skills in late adulthood is the one from Domaneschi and Di Paola (2019). In their study, the authors employed a word-by-word self-paced reading time paradigm to investigate how presupposition processing unfolds in aging, both online and offline. A group of younger participants (mean age = 22.47 years) and a group of older adults (mean age = 63.6 years) were asked to read presupposing sentences and to respond afterward to questions that verified the content of the presuppositions. The target presupposing sentences contained either definite descriptions with a genitive construction (e.g., *the pianist of the pub*) or change-of-state verbs (e.g., *to stop*) and were presented either within a context that satisfied the presupposition or within a neutral context that required accommodation. In addition, since working memory is notably compromised in aging (e.g., Bopp and Verhaeghen 2005; Cappell et al. 2010) and it has been called for in relation to presupposition recovery (Domaneschi et al. 2014), participants’ working memory was measured too. The results revealed patterns of decay. First, during online processing, older participants exhibited longer reading times than the younger group with change-of-state verbs, at the word following the computational point (i.e., the word following an expression such as *X stopped to buy cigars*). The authors interpreted older adults’ higher processing costs as reflecting a peculiar difficulty with change-of-state verbs associated with the extra cost for processing the more complex mental representation of temporally displaced events. Second, in the offline verification task, while participants’ accuracy rates were at ceiling in both groups, age-related effects were present in the response times to the verification questions: as compared to the younger group, older adults were slower at recovering (i) the presuppositions triggered by definite descriptions and (ii) the presuppositions activated by change-of-state verbs when accommodation was involved (vs. satisfaction). The authors interpreted this pattern as suggesting, overall, that presupposition recovery seems to be compromised in normal aging. However, the decline seems bound to the type of trigger as well as to the contextual availability of the presupposition. Recovering a presupposition triggered by definite descriptions appears to be more cognitively demanding and the extra cost might be due to the inferential search for a suitable antecedent in the previous context. Conversely, change-of-state verbs are a lexical trigger whose presupposition is derived via a direct logical implication (Zeevat 1992) and this might facilitate the retrieval in a task that targets the recovery of the presupposition. Nonetheless, recovering a presupposition of change-of-state verbs that was previously accommodated remains costlier in late adulthood. Therefore, contextual availability influences elders’ recovering of the presupposition of change-of-state verbs—even if they seem overall less demanding than definite descriptions. Third, interestingly, Domaneschi and Di Paola (2019) found that working memory predicted presupposition recovery and, also, contributed explaining the patterns of decay. This depended both on (i) trigger type and (ii) contextual availability. First, working memory facilitated participants’ recovery of the presuppositions triggered by change-of-state verbs; on the contrary, with definite descriptions, even participants with a high working memory performance were still slower at presupposition recovering compared to their younger counterparts. More interestingly, working memory modulated presupposition recovery differently in the two age groups depending on condition and trigger type: even older adults with higher working memory scores still were slower than younger participants with better working memory at recovering an accommodated presupposition of definite descriptions. According to the authors, this finding suggests not only an involvement of working memory in the ability to recover presuppositions, but also that the age-related decline in working memory partially accounts for the deterioration of presupposition retrieval during normal aging.

In sum, Domaneschi and Di Paola’s (2019) results provide preliminary evidence on presupposition processing in late adulthood. Their study shows that the ability to update the mental model with presupposed information remains unaffected with aging. Yet, aspects of processing are compromised indeed, and these seem associated with the peculiarities of the triggers. Change-of-state verbs slow processing presumably because of the more demanding nature of their mental representation; definite descriptions increase the cognitive cost likely because of the search for a suitable antecedent. Finally, the patterns of variation in late adulthood appear to hinge on a decline in working memory. No other evidence is available on how presuppositional skills vary with normal aging. As a result, our knowledge of the topic is still very scant and further investigation is worth conducting to better characterize the patterns of decay brought to light by Domaneschi and Di Paola (2019).

### The present study

The main goal of the present study is to extend the findings provided by Domaneschi and Di Paola (2019) and further characterizing presuppositional skills in late adulthood. Without directly manipulating cognitive workload, the results from Domaneschi and Di Paola indicate that working memory plays a role in presupposition recovery. For this reason, we investigated which components of presupposition processing decline with normal aging—if online processing and/or the offline recovery of presupposed information—with a closer look at the direct role of working memory as well as its interaction with the linguistic factors known to impact presupposition cognitive load, namely contextual availability and trigger type.

To do so, we followed Domaneschi and Di Paola (2019) and used a reading time paradigm^7^ in which a group of older and a group of younger participants read stories containing presupposition triggers and afterward responded to verification sentences about the content of the presupposition. The target sentences of the stories could contain either a change-of-state verb (*stop* or *begin*) or a definite description and were presented either within a supporting context that satisfied the presupposition or within a neutral context that elicited accommodation. Crucially, we manipulated the mental workload: participants read the presupposing stories in a condition of low or high mental workload. These were obtained by manipulating the amount of information provided in the stories, such that more distractor sentences were included in the stories when the mental load was high to tax participants’ working memory. We collected reading times as well as accuracy and response times to the verification sentences.

As Table 1 depicts, in our study, we not only manipulated the contextual availability—neutral vs. supported—but also the cognitive load.^8^ To better understand the impact of cognitive load, we used stories in which the cognitive load was comparable to the one in Domaneschi and Di Paola’s study as well as a condition in which the cognitive load was high, i.e., five distractor sentences were part of these stories. Most important, the target sentence appeared in a segment-by-segment sequence—and segmentation already started at context sentence 2—and not in a word-by-word sequence as this was the case in Domaneschi and Di Paola’s study. It is also worth mentioning that the target sentences of the conditions containing a definite description involved a more complex genitive construction such as *the pianist of the pub* in Domaneschi and Di Paola’s study, while in the present study we used a simpler referential expression such as *the swimming pool* to avoid that the cognitive load manipulation is tainted by the complexity of the target sentence (i.e., sentence comprehension becomes more demanding with age in more complex sentence constructions, e.g., Stine-Morrow et al. 2000).

**Table 1:**
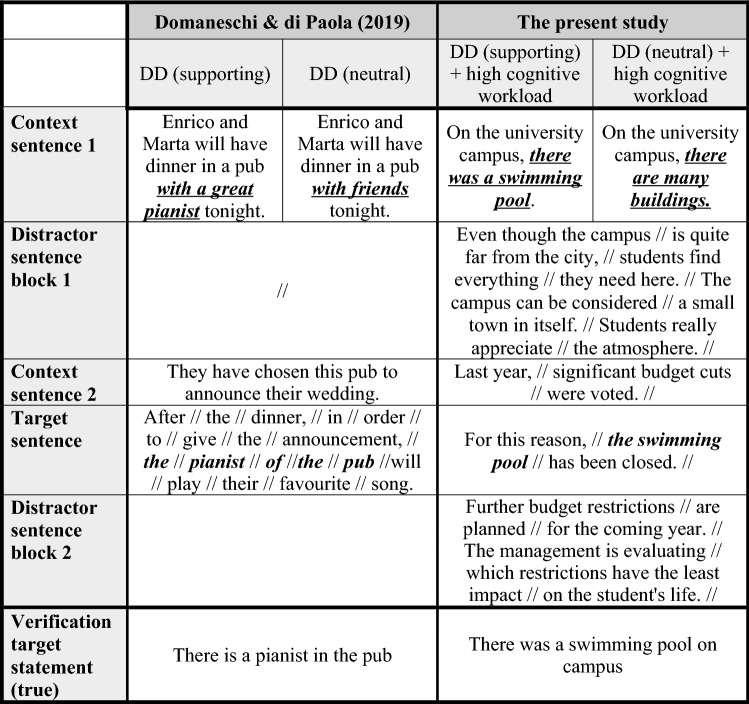
Comparing the design and materials used by Domaneschi & Di Paola with the present study

Each // indicates that a new screen unfolds

*DD* Definite descriptions

Assessing presuppositional skills with a paradigm that includes both an online and an offline task is essential to disentangle which aspects of presupposition comprehension decline with aging, if processing per se and/or recovery. In turn, within a life-span perspective, this is very informative since processing and recovery reflect distinct processes. Processing as measured by reading times informs us about the cognitive costs that underlie presupposition understanding during online language comprehension, while the sentence unfolds. Differently, recovering the presupposed content informs us about the ability to (i) retrieve information from the discourse mental model that has been conveyed as presupposed and (ii) update the discourse mental model with the presupposed content. In a verification task, (i) is measured by response times that, therefore, provide an indication of the cost of the recovery process; (ii) is measured by participants’ accuracy, that constitutes a measure for the effective updating of the mental model. Additionally, since previous studies revealed that both contextual availability and trigger type impact the cognitive load of presupposition comprehension, keeping these two factors in the design is essential. Last but not least, focusing on the mental workload is crucial too. In fact, this allows for a more fine-grained examination of the role of working memory in presupposition processing in general, as well as in its patterns of decay. The present study aims to provide answers to the following research questions:

1. Does the manipulation of cognitive workload in online comprehension of presuppositions involve higher processing costs for healthy older adults in comparison to younger ones?
2. What is the impact of cognitive workload in presupposition recovery in healthy older and younger adults?
3. Does the type of the change-of-state verb, i.e., *begin* vs *stop* have an impact on the ability to update the discourse mental model with presupposed information?

First, as pointed out in the introduction, online processing of presuppositions declines with age (Domaneschi and Di Paola 2019). Furthermore, Domaneschi and Di Paola’s results indicate that working memory capacity has an impact on the recovery of presuppositions, i.e., higher responses times to the verification statements when working memory capacity decreases. The present study, therefore, not only examines the impact of cognitive load during the recovery of presuppositions, but also during its online processing. Since information processing speed and inferential processing abilities are more compromised in older adults (e.g., Myerson et al. 1992; Stine and Hindman 1994), we expect that the higher cognitive load condition leads to a greater slowdown of the presupposed content compared to the low cognitive load condition in older vs younger adults.

Second, based on Domaneschi and Di Paola (2019), we expect patterns of decline especially in the ability to recover presupposed information. Therefore, older adults should exhibit longer response times than younger adults in the verification task. Moreover, based on Domaneschi et al. (2014) and Domaneschi and Di Paola (2019), the recovery of background information should be generally affected by the mental workload. Importantly, however, age-related differences should emerge when the mental workload interacts with contextual availability. In other words, since previous studies showed that accommodation is costlier than satisfaction and working memory has been called for with regards to this, recovering a presupposition previously accommodated should be even costlier for older adults supposedly because of compromised working memory capacities. Therefore, the extra costs should emerge more clearly when the mental workload is manipulated.

Third, to the best of our knowledge, this is the first study that explores the processing of more than one change-of-state verb. For this reason, we exploratorily analyze whether there is a processing difference between the change-of-state verbs *stop* vs. *begin* and how these two verbs are in general processed across the life span.

## Methods

### Participants

Two groups of native French speakers participated in the experiment: a group of 25 younger adults (18 females; *M*_age_ = 23, *SD*_age_ = 5.5; *M*_schooling_ = 12 years, *SD*_schooling_ = 0) and a group of 23 older adults (19 females; *M*_age_ = 65.43, *SD*_age_ = 2.35; *M*_schooling_ = 11.86 years, *SD*_schooling_ = *1.65*). The group of younger adults was composed of students recruited from the University of Neuchâtel. The group of older participants was recruited from the University of the 3^rd^ age of Neuchâtel and seniors’ organizations operating in the region of Neuchâtel, such as ProSenectute. Participants in both groups had normal or corrected to normal vision; they showed no history of neurological or psychiatric disorder, no major head trauma, and did not receive any neuroactive medication in the preceding 6 months.

The group of older adults was screened for general cognitive impairment as well as for potential language impairments. The former was assessed using the French adaptation of the Montreal Cognitive Assessment battery (MoCa; Nasreddine and Patel 2016; Nasreddine et al. 2005); the latter was assessed with the *Detection Test for Language Impairments in Adults and the Aged* (DTLA, Macoir et al. 2017), which provides a quick, sensitive and standardized test to screen for language disorders. All participants in the older group scored above the threshold of 26 in the MoCa assessment (out of 30; *M*_MoCA_ = 28.30, *SD*_MoCA_ = 1.74), therefore showing uncompromised cognitive performance. All participants scored above the threshold of 85 in the DTLA test (out of 100, *M*_DTLA_ = 98.04, *SD*_DTLA_ = 4.72), thus revealing no signs of language impairment. Written informed consent was obtained from every participant prior to the beginning of the experiment.

The present study was approved by the Ethical Committee of the University of Neuchâtel (reference number 34/2018).

### Materials

#### Experimental stimuli

The experimental material was composed of a final set of 80 quadruplets of stories in French containing a presupposition, i.e., four different versions of each story that corresponded to each of the experimental conditions. Each story included a *context sentence 1*, a *context sentence 2*, and a *target sentence*. The target sentence could contain one of two presupposition triggers, namely a definite description or a change-of-state verb. Two change-of-state verbs were used, *arrêter* (to stop) and *commencer* (to begin).^9^ Items were presented in two contexts, *satisfaction* (SAT) and *neutral* (NEU). These were obtained by manipulating context sentence 1 that it could provide either a supporting context that satisfied the presupposition of the target sentence (i.e., SAT), or a neutral context that did not satisfy the presupposition and therefore required accommodation (i.e., NEU). Moreover, stories in both contexts were presented in two conditions of mental workload, *Low* versus *High*. These were obtained by varying the quantity of information provided in the stories. In the condition of low mental workload, each story was composed of two context sentences and the target sentence. In the high mental workload condition, 5 distractor sentences were added to each story. These were located between Context sentences 1 and 2 (i.e., distractor sentences 1 to 3) and after the target sentence (i.e., distractor sentences 4 and 5)—see Table 2 for a sample story containing a definite description. After each story, three true/false verification statements were presented: one *target verification sentence* that assessed the presupposed content triggered in the target sentence of the story, and two distractors. The target verification sentence was always true; the number of true and false responses was counterbalanced.

**Table 2:**
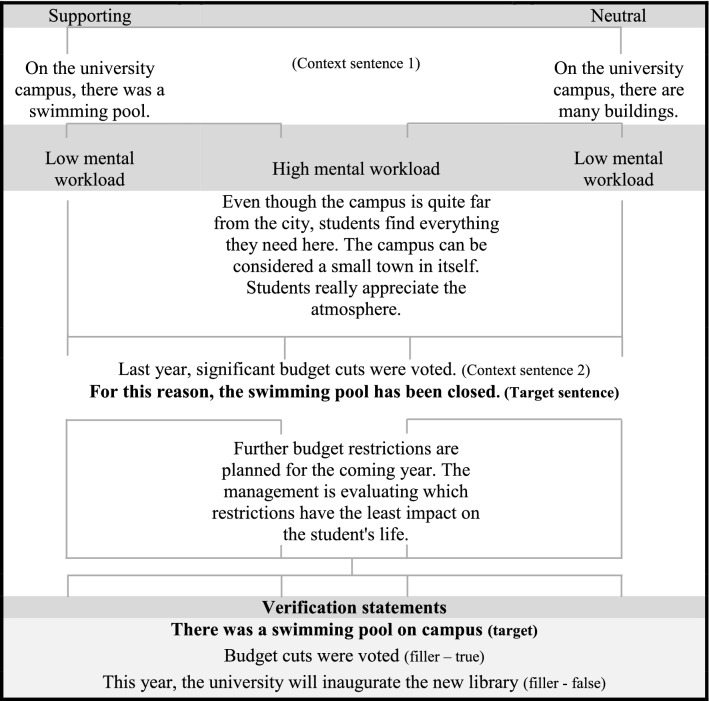
Sample story for definite descriptions (DD) in the supporting and neutral condition

A sample story in French for DDs and CSVs is shown in the Appendix (Tables 4, 5)

To introduce some distraction, we added 40 coherent filler stories, which included a context sentence 1, a context sentence 2, and a target sentence. Additionally, in half of the cases we added a distractor sentence that followed the target sentence.

##### Pre-testing of the experimental material

To obtain the final set of the 80 story quadruplets, we proceeded as follows. Two norming studies were first conducted on the stories in the neutral context to ensure these were acceptable both in the long and short version of, respectively, the high and low mental workload conditions. This procedure led to the selection of the final story items in the neutral context, for both DDs and CSVs, and in both high and low mental workload condition. Based on this, the story counterparts in the satisfaction context were then created. In what follows, we provide the most relevant details of the norming studies as well as the most relevant results.

Both studies were conducted on the online survey platform Qualtrics. Participants were asked to rate stories involving a not satisfied presupposition on a 5-point Likert scale ranging from (1) totally unacceptable to (5) fully acceptable. Informed consent was obtained from all participants.

For the first norming study, 334 students from the University of Neuchâtel (*M*_age_ = 23.31, *SD*_age_ = 4.97) were asked to rate the acceptability of an initial set of 50 stories with DDs—there were two versions for each story, i.e., one for the low mental workload condition and one for the higher one—and 46 stories with CSVs—there were also two versions for each story—plus a set of 6 filler stories (three acceptable stories and three unacceptable ones). The questionnaire was pseudo-randomized: (i) no condition appeared more than twice consecutively and (ii) the participant was assigned either to the long or to the short version of a given story but did not see both versions of the same story. Each participant read 16 stories in total (i.e., 5 short neutral stories, 5 long neutral stories, and 6 filler stories).

Non-native French speakers and participants who rated at least one unacceptable filler story higher than 3 (i.e., more or less acceptable) or at least one acceptable filler story lower than 3 were excluded from statistical analysis. Based on these criteria, data from 232 participants (*M*_age_ = 22.9, SD_age_ = 4.58; 174 F, 54 M, 4 others) were analyzed. Several *t* tests were carried out to conduct an item analysis that compared the acceptability rating of each story in low vs. high mental workload. The story pairs (i.e., high vs. low mental workload) that significantly differed in acceptability (*p* < .05) and those in which one or both stories received a mean acceptability score lower than 2.5 were modified and normed again. After pretest 1, 21 DD story pairs and 34 CSV story pairs did not show a significant difference. Therefore, these stories were kept. The story pairs that showed a significant difference were modified and their acceptability was assessed in a second norming study.

The second norming study was conducted on 149 students from the University of Neuchâtel (*M*_age_ = 23.92, *SD*_age_ = 5.25), different from pretest 1. For data analysis, the same exclusion criteria and statistical procedure as before were used. Based on this, data from 124 participants (*M*_age_ = 23.81, *SD*_age_ = 5.1; 92 F, 31 M, 1 others) were analyzed.^10^

The final set of experimental stimuli in the neutral context for both presupposition triggers—DD and CSV—was composed of 40 story pairs, i.e., one version for each mental workload—low and high. The analysis of the acceptability rating scores indicated that there was neither a significant difference between the low versus high mental workload condition for definite descriptions [*t*(1126) = −0.87421, *p* = .3832; *M*_low_mental_workload_ = 3.471 (*SD*_low_mental_workload_ = .43); *M*_high_mental_workload_ = 3.53 (*SD*_high_mental_workload_ = .54)] nor for both versions of the CSV stories [*t*(952) = 0.0878, *p* = .93; *M*_low_mental_workload_ = 4.01 (*SD*_low_mental_workload_ = .47); *M*_high_mental_workload_ = 4.00 (*SD*_high_mental_workload_ = .39)].

##### Setup of lists for the experiment

Our final set of 80 stories, i.e., 40 stories involving a definite description and 40 involving a change-of-state verb were randomized in such a way that each participant would only see one of each scenario. Therefore, four different lists were created. Given our 2 × 2 × 2 (contextual availability × trigger type × cognitive load) experimental design, each participant saw in total 10 stories of each experimental condition. In total, each of the four lists contained 120 stories, i.e., 80 experimental stories and 40 filler stories. Participants were randomly assigned to one of the four lists.

### Experimental procedures

The experiment was a 4 × 2 mixed Latin square design, with Age Group (Younger vs. Older) as the between-subjects variable, and Trigger Type (DDs vs. CSVs), Context (SAT vs. NEU) and Mental Workload (High vs. Low) as the repeated measures. As measures of online processing, we collected participants’ reading times (RTs) on two regions of interest of the target sentence (i.e., segments 2 and 3), as well as the RTs of segment 1 and the total RTs for the whole sentence (i.e., TOT). Concerning the second and third segment of the target sentence, these two segments refer to what has been identified as the triggering point—segment 2—and the computation point—segment 3 (see Tiemann et al. 2011). According to Tiemann et al. the triggering point refers to the point of the sentence where the presupposition trigger appears—e.g., *John stopped*—and the computation point refers to the point where the presuppositional content becomes in fact available, e.g., *smoking cigars* (see Table 3, target sentence of the change-of-state-verb). As measures of offline processing, we collected participants’ accuracy (i.e., correct responses to target verification statements) and response times to the verification statements.

**Table 3:** Segment-by-segment presentation of the target sentences

Trigger	Segment 1	Segment 2	Segment 3
DD	Pour cette raison,	la piscine	a été fermée.
	*[For this reason,*	*the swimming pool*	*has been closed.]*
CSV	Pour cette raison,	Jean a arrêté de	fumer des cigares.
	*[For this reason,*	*John stopped*	*smoking cigars.]*

Participants sat in a quiet room facing a computer screen and were instructed to read the visually presented stimuli. Each experiment started with two trial stories to familiarize participants with the task and procedure. Afterward, the experiment started. Stimuli were presented in black font centered on a white background. At the end of the last verification statement of each trial, the following instruction appeared on the screen ‘*End of story. A new story will unfold. Please press the space bar*.’ After the participant hit the space bar, a new story began. The first sentence—context sentence 1—was always presented as a whole, whereas the rest of the story was presented in a segment-by-segment self-paced reading paradigm. At the end of each story, the participant had to answer the three verification statements. After 20 stories, the participant was invited to take a short break and as soon as she felt ready to continue the experiment, a new block of 20 stories began. In total, the 120 stories were divided into 6 blocks of 20 stories each. On average, the younger participants took one hour to complete the experiment and the older adults took around one hour and a half. The MoCA and the DTLA tests were administered to the older group at the end of the session to avoid any fatigue bias during the main experiment. At the end of the experiment, participants were debriefed about the purpose of the present research.

### Statistical analyses

The reading times of each sentence region (i.e., segments 1–3 and the total RTs) as well as the response times to the verification statements were analyzed with linear mixed models statistics (LMMs), using the *lme4* (Bates et al. 2015) and the *lmerTest packages* in the R environment. The random effects structure was kept constant for all statistical models. To overcome model non-convergence, the final random effects structure was a parsimonious one that included random intercepts for subjects and items and by-subjects random slope for Context. This was obtained on the basis of the following criterion: starting from the maximal random effects structure given the experimental design,^11^ the LMM was simplified, in a backward fashion, by removing one random effect at a time, until convergence was met (Barr et al. 2013; Matuschek et al. 2017). The fixed effects structure of the LMMs included Age Group, Context, Trigger Type, Mental Workload, and the resulting interactions. We report Analysis of Variance results obtained with the *lmerTest package* for the LMMs.

Accuracy to the verification statements was coded 1 for correct responses and 0 for incorrect responses. The statistical analysis was carried out using a Generalized Linear Model (GLM) with a logit link function.^12^

## Results

In what follows, we report the relevant results. See Tables 6, 7, and 8 in Appendix for details of all statistical analyses.

### Reading times on target sentences

To answer the first research question—*Does the manipulation of cognitive workload in online comprehension of presuppositions involve higher processing costs for healthy older adults in comparison to younger ones?*—we analyzed the overall reading times as well as the reading times of all three segments of the target sentence of the self-paced reading paradigm.

**Fig. 1:**
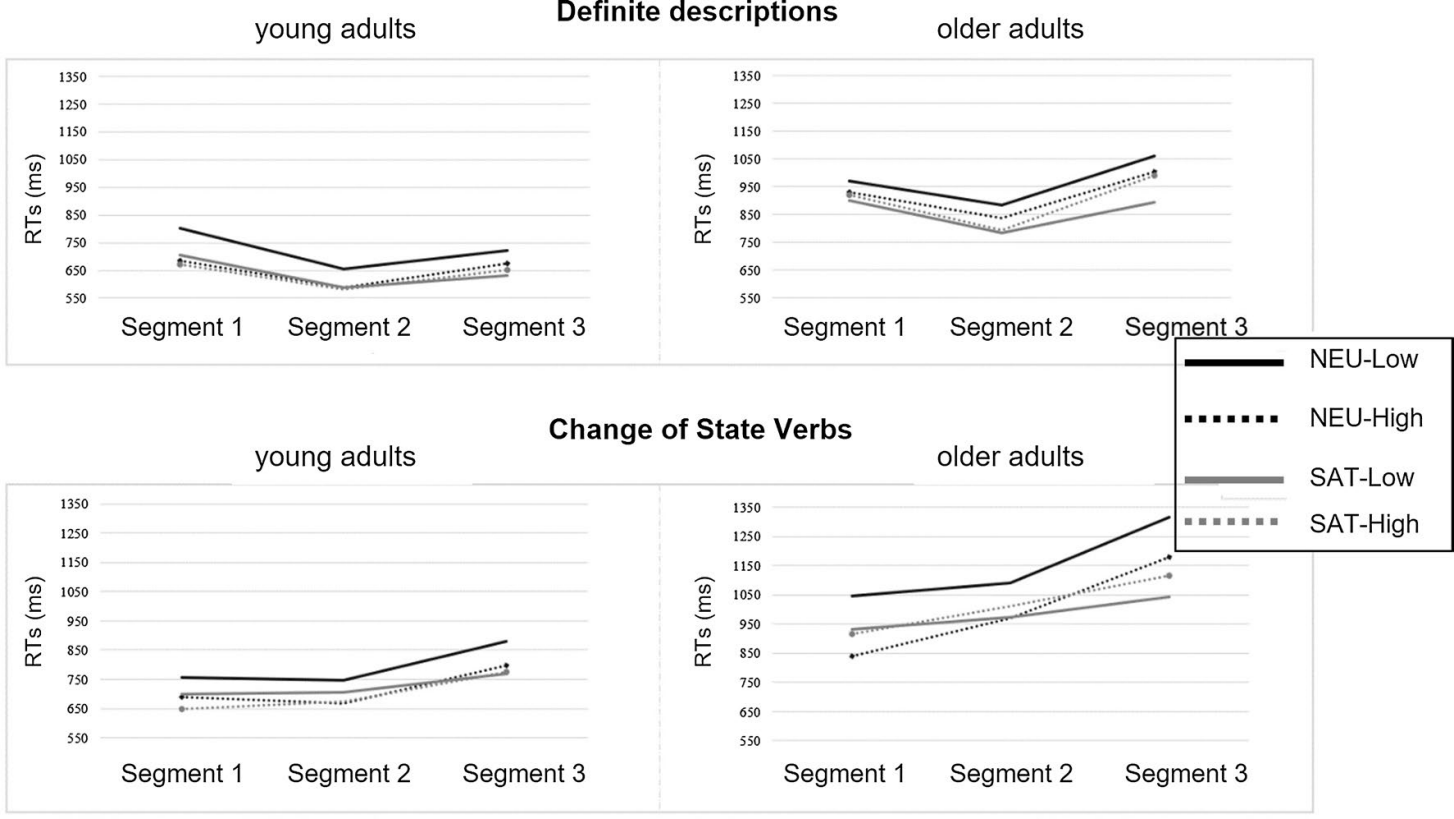
Reading times (RTs) at all three segments for definite descriptions (DDs) and change-of-state verbs (CSVs). Top left: RTs of young adults for DDs; Top right: RTs of older adults for DDs; Bottom left: RTs of young adults for CSVs. Bottom right: RTs of older adults for CSVs

The mean RTs for the regions of interest are depicted in Fig. 1. Overall, statistical analyses revealed a range of main effects as well as a range of interaction effects. For clarity of exposition, we report these separately.

#### Main effects

The group of older adults was overall slower than younger adults as suggested by a main effect of Age Group at all sentence regions [Segment 1: *F*(1, 45.9) = 13.52, *p* < .0001); Segment 2: *F*(1,45.7) = 39.74, *p* < .0001; Segment 3: *F*(1,45.7) = 24.29, *p* < .0001; TOT: *F*(1,45.8) = 24.82, *p* < .0001]. Moreover, participants were slower at reading presupposing sentences when these were presented in the neutral context (vs. supporting), as suggested by a main effect of Context at all sentence regions [Segment 1: *F* (1, 239.1) = 4.02; *p* = .04; Segment 2: *F*(1,259) = 5.94; *p* = .01; Segment 3: *F*(1,228.4) = 10.74; *p* = .001; TOT: *F*(1,258.4) = 8.95; *p* = .003]. Interestingly, our participants’ RTs were also slower in the condition of low (vs. high) mental workload at Segments 1 [*F*(1, 308) = 10.08; *p* = .001) and 2 (*F*(1,308.6) = 5.14; *p* = .02]. This effect dissolved at Segment 3 [*F*(1, 311.4) = .33; *p* = .56], but re-emerged later on in the total RTs [*F*(1,309) = 4.36; *p* = .03]. Finally, participants of both groups took overall longer reading the target sentences when the presupposition was triggered by CSVs than DDs: a main effect of trigger type emerged first at Segment 2 [*F*(1,308.5) = 67.39; *p* < .0001; Segment 1: *p* = .71], persisted at Segment 3 [*F*(1,311.4) = 30.61; *p* < .0001], and was also presented in the total RTs [*F*(1,308.9) = 24.29; *p* < .0001].

#### Interaction effects

**Fig. 2:**
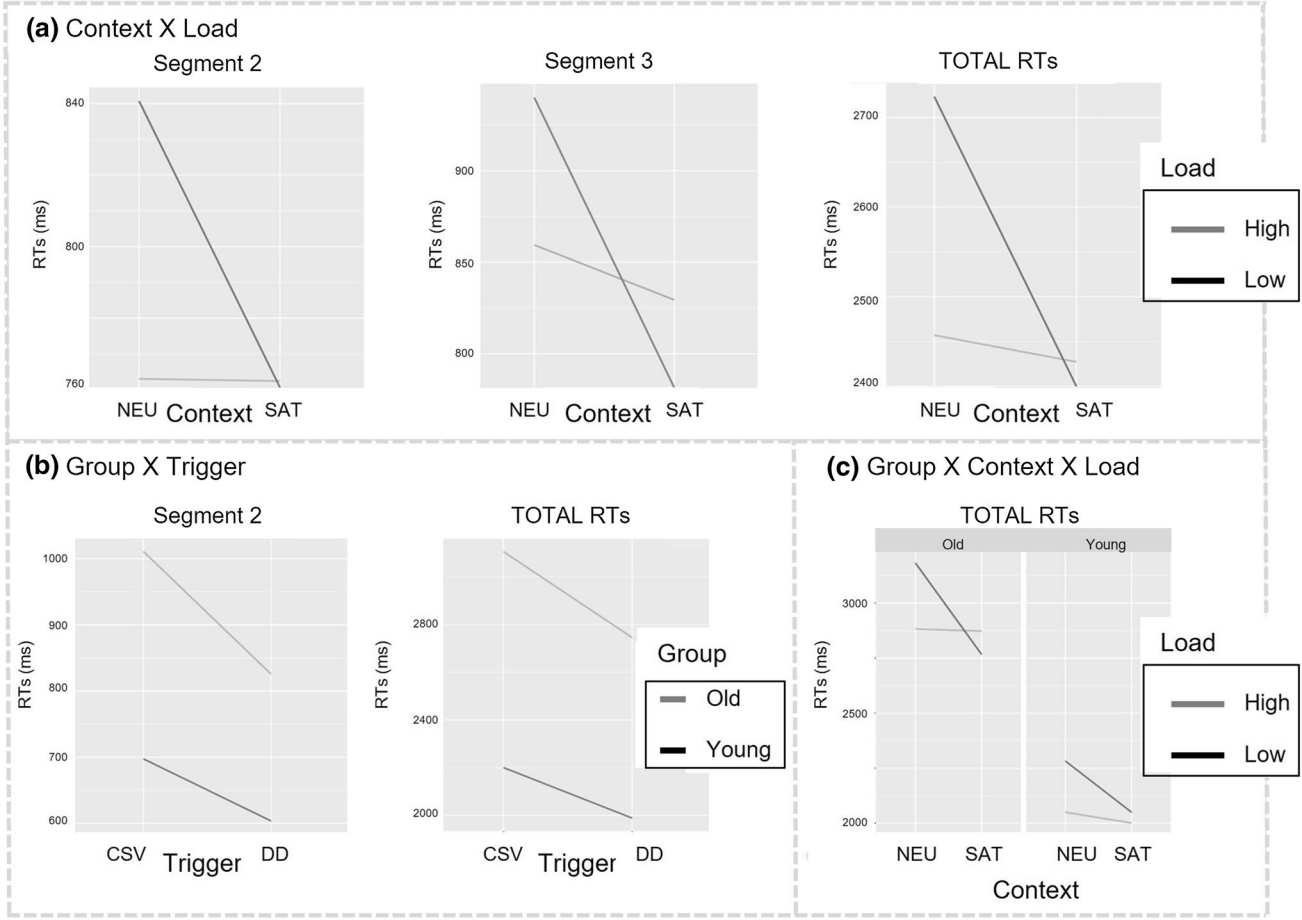
Interaction effects for the reading times (in ms) across regions of interest (Segment 2, Segment 3 and total reading times). **a** Interaction effects between Context and Load at Segments 2 and 3 as well as for total RTs; **b** Interaction effects between Group and Trigger at Segment 2 and for total RTs; **c** Interaction effect between Group, Context, and Load on the total RTs

Most important, the LMMs statistics revealed a range of significant interactions, too. First, at all sentence regions, the interaction between Context and Load was significant, indicating that participants’ RTs in the low mental load condition (vs. high mental load) were higher when the context was neutral as compared to when it was supporting [Segment 1: *F*(1,307.9) = 4.92; *p* = .02]; Segment 2: [*F*(1,308.6) = 6.30; *p* = .01] Segment 3: *F*(1,311.4) = 6.48; *p* = .01; TOT: *F*(1,308.9) = 7.64; *p* = .006) (Fig. 2a).

Second, some significant age-related interactions emerged. Even if older adults were slower than younger adults at processing both presupposition triggers, they were even more so when CSVs were involved, as revealed by a significant interaction between Group and Trigger at Segment 2 [*F*(1, 3413) = 22.21; *p* < .0001] (Fig. 2b). This interaction effect remained in the total RTs [*F*(1, 3443) = 8.59; *p* = .003], together with a 3-way interaction between Group, Context, and Load [*F*(1, 3443) = 4.64; *p* = .03] that suggests that older adults were particularly slower in the neutral context when the mental load was low (vs. highly) taxed (Fig. 2c).

### Verification statements: accuracy and response times

To answer the second research question—*What is the impact of cognitive workload in presupposition recovery in healthy older and younger adults?*—we analyzed the accuracy scores and response times to the verification questions.

**Fig. 3:**
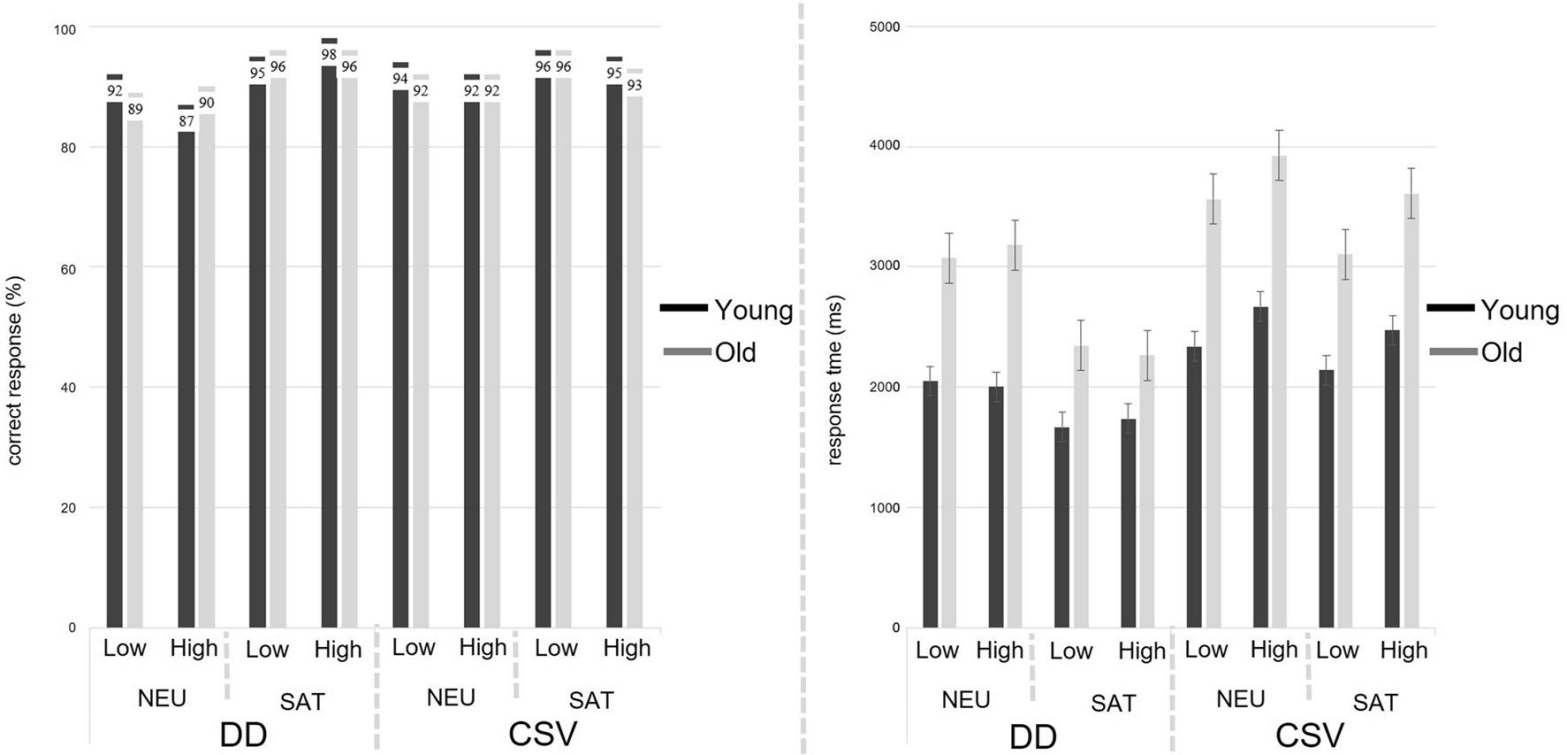
Left: Accuracy to verification statements by age group and condition for DDs and CSVs. Right: Response Times: mean response times (ms) to verification statements by age group and condition for DDs and CSVs. *NEU-Low/High*: Neutral context and Low/High Mental Workload; SAT-Low/High: Satisfaction context and Low/High Mental Workload

The younger and older group’s accuracy scores and response times to the target verification sentences are depicted in Fig. 3. Starting with accuracy, as Fig. 3 (left panel) shows, both groups were highly accurate in all conditions of the verification task and the GLM statistics revealed no significant main effects nor interaction effects (all *p*_*s*_ = n.s).

**Fig. 4:**
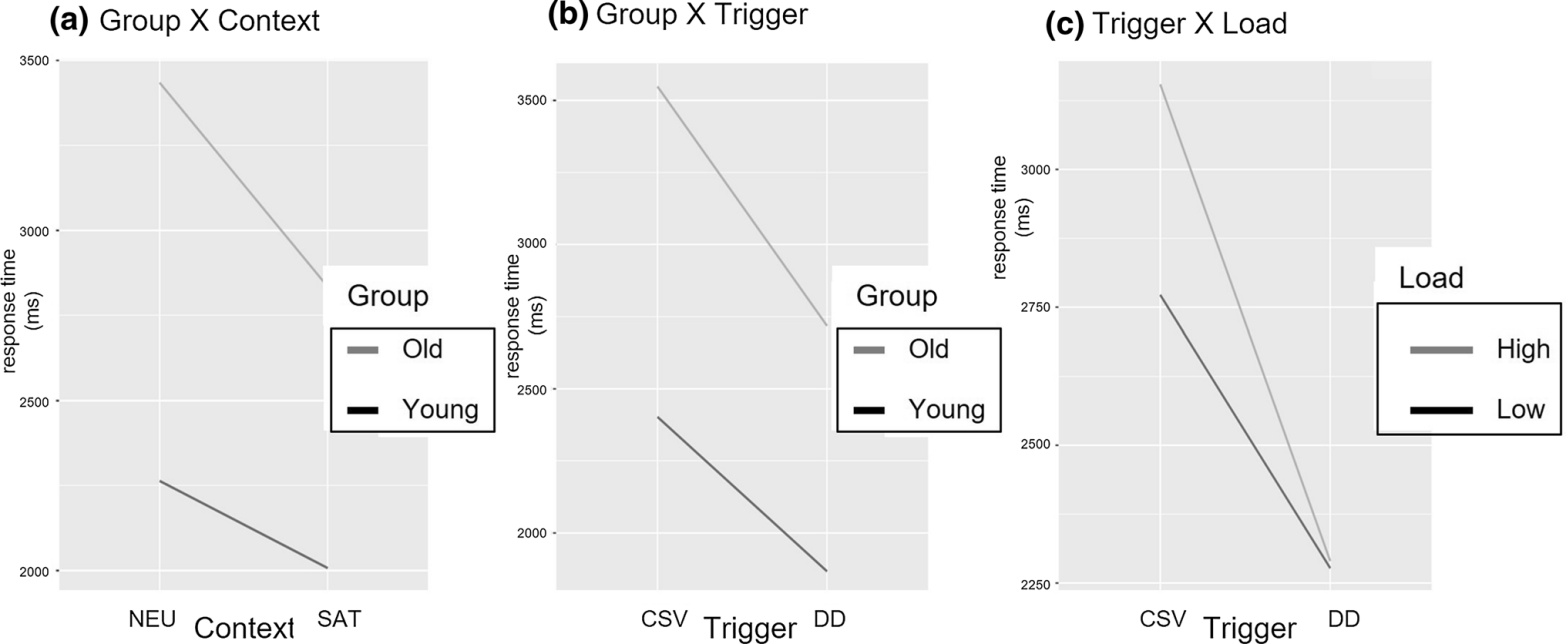
Interaction effects for response times (ms). An interaction between **a** group and context; **b** group and trigger; and **c** trigger and load

As for response times, Fig. 3 (right panel) shows that the older participants were substantially slower in responding compared to the younger participants. Response times of all responses to the verification task were analyzed. LMMs statistics revealed not only a significant main effect of Age Group [*F*(1,43.81) = 31.59; *p* < .0001], but also a significant main effect of Context [*F*(1,166.81) = 14.69; *p* = .0001] and of Trigger Type [*F*(1,308.92) = 45.40; *p* < .0001]. In addition, the main effect for Mental Workload approached significance [*F*(1,308.95) = 3.78; *p* = .053]. More interestingly, a range of interaction effects emerged too, both age-related and associated with the mental load. First, the interaction between Group and Context was significant [*F*(1,43.30) = 5.21; *p* = .02], revealing that the increase in response time in the neutral versus the satisfied condition was higher for older adults than for younger adults (Fig. 4a). Second, a significant interaction between Group and Trigger [*F*(1,2309.67) = 8.40; *p* = .003] indicated that older adults were slower than younger adults responding to verification statements that assessed the presupposition triggered by CSVs than DDs (Fig. 4b). Finally, the interaction between Load and Trigger approached significance [*F*(1,308.92) = 3.36; *p* = .067], thus suggesting that both age groups tended to be slower in the high than in the low mental workload condition when CSVs (vs. DDs) were involved (Fig. 4c).

In sum, results on response times revealed that older adults were slower responding to verification statements that verified presuppositions previously accommodated and that were triggered by change-of-state verbs. Also, when the mental load was highly taxed, participants’ response tends to decrease with change-of-state verbs as compared to definite descriptions.

#### *Stop *versus *begin:* accuracy and response times

To answer the third research question*—Does the type of the change-of-state verb, i.e., begin vs stop have an impact on the ability to update the discourse mental model with presupposed information?*—for the CSV *stop* vs. *begin,* the accuracy scores and response times to the verification statements were analyzed.

Since two CSVs were used (i.e., *to stop* and *to begin*) and the response times results (see previous section) showed slowdowns associated with CSVs, a second analysis was conducted on accuracy and response times for CSV trials to investigate any effect with respect to the two change-of-state verbs (i.e., *begin* (*commencer*) and *stop* (arrêter)).^13^

Participants of both groups correctly responded at ceiling to verification sentences for both *arrêter* and *commencer* across all experimental conditions. No significant main effects nor interactions emerged from the GLM statistics.

**Fig. 5:**
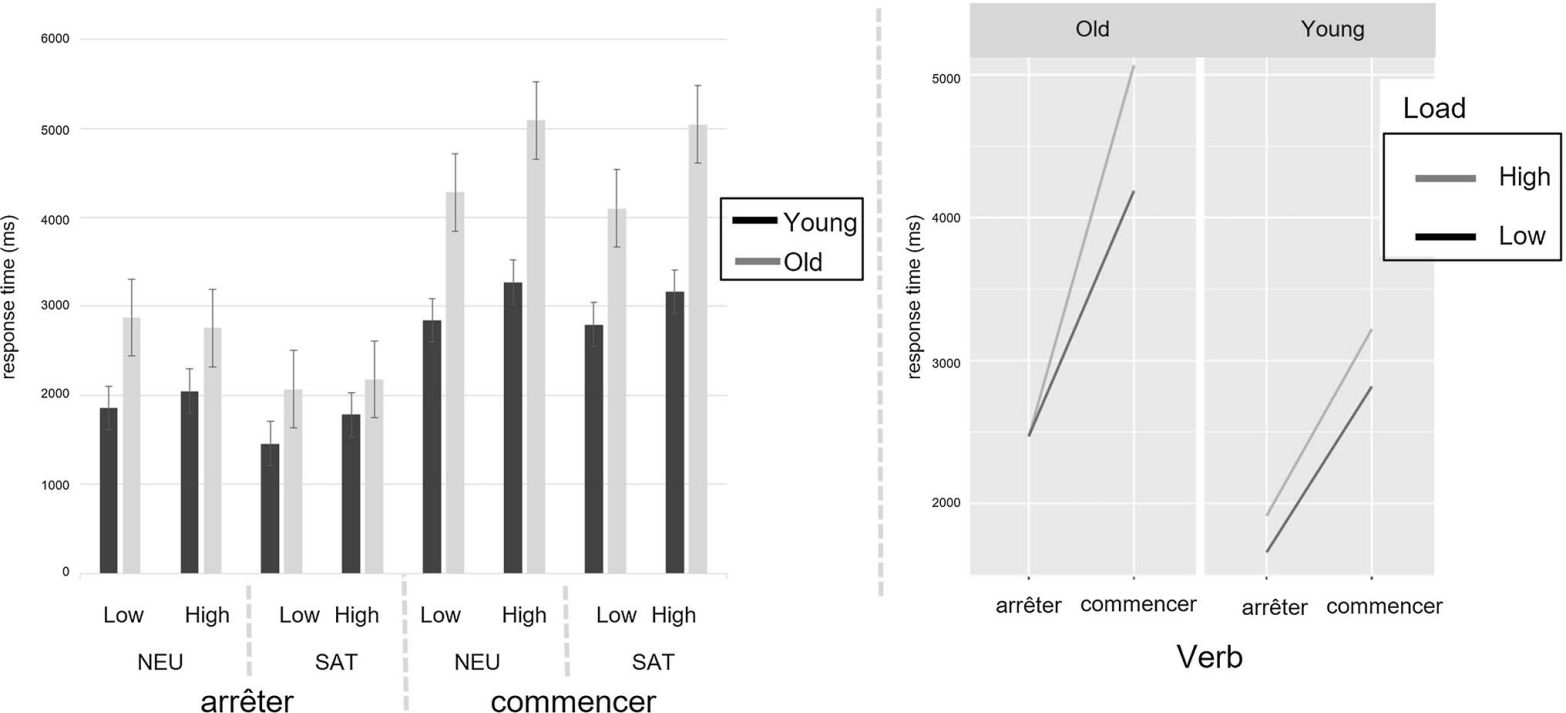
Left: mean response times (ms) for the change-of-state verbs *arrêter* and *commencer* by age group and experimental condition; *NEU-Low/High* Neutral context and Low/High Mental Workload; SAT-Low/High Satisfaction context and Low/High Mental Workload. Right: Interaction effect plot between Group, Load, and Verb Type

The mean response times for both groups across verb types and experimental conditions are depicted in Fig. 5 (left panel). LMMs statistics revealed significantly higher response times for *commencer* than *arrêter*, as suggested by a main effect of verb type [*F*(1,145.89) = 296.75; *p* < .0001]. Most interesting, a range of significant interactions also emerged. First, the increase in response time for *commencer* versus *arrêter* was higher for older than younger adults [interaction between Group and Verb Type: *F*(1,2420.67) = 34.03; *p* < .0001]. Second, the increase in response time in the neutral versus supporting condition was significantly higher for *arrêter* than *commencer* [interaction between Context and Verb Type: *F*(1, 145.92) = 4.32; *p* = .04]. Third, the increase in response times in high (vs. low) mental workload was higher for *commencer* than for *arrêter* [interaction between Load and Verb Type: *F*(1, 145.89) = 6.66; *p* = .01]. Finally, and even more interestingly, the interaction between Group, Load, and Verb Type was significant too [*F*(1,2420.67) = 5.51; *p* = .02], suggesting that the interaction between Load and Verb Type depended on age group: the response time increase for *commencer* versus *arrêter* in High versus Low Mental Workload was higher for older adults than it was for younger adults (Fig. 5right panel).

## Discussion

The main goal of this study was to extend the only currently available study about presupposition processing across the life span by Domaneschi and Di Paola (2019) and shed more light on which component of presupposition processing declines in late adulthood. In particular, we focused on the direct role of working memory, that is notably compromised with aging and that was found to partially account for the aging-related decline of presuppositional skills. Our participants read presupposing sentences that contained either definite descriptions or change-of-state verbs within a supportive or a neutral context, and in conditions of low vs. high mental workload. After reading the stories, participants responded to verification sentences about the content of the presuppositions. We measured the reading times as well as the accuracy and response times to the verification task; the former being a measure of online processing, the latter providing measures for presupposition recovery. Our results of the reading time paradigm indicate the following:

1. Older participants’ reading time is in general slower than the reading time of younger participants (Main Age effect)
2. Older participants’ reading time increase during the second and third segment of the comparison between change-of-state verbs and definite descriptions is higher than this is the case for younger participants (Group X Trigger interaction).

Moreover, the principal results of the verification task indicate the following:

1. Recovering a presupposition in neutral contexts is more difficult for older adults than for younger adults (Group X Context interaction). More precisely, the age-related slowdown in recovering a presupposition is higher in neutral contexts than this is the case for supporting contexts.
2. Recovering presuppositions of change-of-state verbs is cognitively more demanding for older adults when compared to definite descriptions (Group X Trigger interaction).
3. In addition to (2), recovering the presupposed content of the change-of-state verb *begin* is in general more demanding for older adults than recovering the presupposed content of *stop* (Group X Verb type interaction). Moreover, this slowdown is even more enhanced in the high mental workload condition (Group X Verb type X Load).

In the first part of the discussion, we will focus on the results of the reading time paradigm. It will also be pointed out that the results on this task revealed some unwanted biases. Thus, we take the reading time results with caution. The results of the offline verification task will subsequently be discussed in more detail.

### Online processing of presuppositions (reading time paradigm)

Results on the reading times of the target presupposing sentences revealed a main age-group effect on all sentence regions, with longer RTs associated with the group of elderly participants. As expected, this suggests a general decline in processing abilities with normal aging and confirms that speed of information processing is compromised in late adulthood (e.g., Stine and Hindman 1994). Most important, a significant interaction between Context and Load emerged both on segment 2 of the sentence (i.e., at the triggering point) and on segment 3 of the sentence (i.e., the computational point). For both segments, participants took longer in reading the relative sentence regions of shorter (vs. longer) stories when the context was neutral (vs. supporting). This suggests higher cognitive efforts in processing presupposition that needs accommodation when the cognitive system is low taxed. This effect might be due to the fact that shorter stories eliciting a low mental workload also provided less information than the longer stories in the condition of higher mental workload. Therefore, it might be the case that if on the one hand participants’ cognitive resources were not taxed by a greater amount of information to process, on the other hand this increased the cognitive effort required to process presuppositions that needed to be accommodated. In other words, the less the amount of information provided by the stories, the greater the inferential work needed to repair a presuppositional failure.

A significant interaction between Group and Trigger emerged on segment 2 of the target presupposing sentences, with older participants’ slower reading times for change-of-state verbs than definite descriptions. Segment 2 was the sentence region that corresponded to the triggering point in this experiment, that is to the trigger itself—a point at which the reader is alerted that the context will have to entail a given proposition for the utterance to make sense. Older adults’ slower reading times on this region therefore indicate an increased effort at processing change-of-state verbs themselves, as compared to definite descriptions. Older adults’ higher processing costs with change-of-state verbs were also found in Domaneschi and Di Paola (2019), even though more forward in the sentence. The fact that in our study the increased processing costs emerged earlier is likely due to a different segmentation of the sentences (i.e., segment-by-segment in the present study and word-by-word in Domaneschi and Di Paola), together with substantial differences in the materials that include shorter stories in Domaneschi and Di Paola. However, beyond this, the trend revealed by both studies is similar: older adults exhibit a slowdown while processing online change-of-state verbs as compared to definite descriptions. Following Domaneschi and Di Paola (2019), such difficulty might be explained by the fact that, being a temporal trigger, change-of-state verbs require the mental representation of temporally displaced events and this might in fact increase the processing effort (see also Domaneschi et al. 2014 and Tiemann et al. 2015 for higher processing costs of temporal triggers).

Significant effects related to the experimental manipulations of this study (i.e., effects of contextual availability and mental workload) emerged also at segment 1 of the presupposing sentences. This is overall unexpected. In particular, the effect associated with contextual availability suggests unwanted experimental biases in the construction of the materials. This represents a limitation of the study and prevents us from speculating too much on the patterns of results emerged from participants’ reading times. Nevertheless, we want to draw attention to an interesting pattern with respect to the reading times of the first segment and the total reading times. In general, participants benefited more from enriched contextual information in the neutral stories than this was the case for the supportive stories (for instance, younger adults’ reading time to the first segment of the target sentence decreased by 117.43 ms for definite descriptions and 68.07 ms for change-of-state verbs in the long neutral condition when compared to the short neutral condition). A similar pattern can be observed for older adults who also benefited from those longer stories in which the presupposition must be accommodated (for definite descriptions, the reading time of the first segment decreased by 40.27 and for change-of-state verbs by 207.67 ms). Interestingly though, these reading time benefits were less prominent, if existent, in the satisfaction condition (see Fig. 1 in Results section). It could be argued that target sentences involving a presupposition that is not yet part of the common ground are more rapidly processed in richer contexts. For instance, previous research has indicated that older adults’ processing time is particularly reduced when information is ambiguous (e.g., Miller and Stine-Morrow 1998). Therefore, overall higher reading times in less rich contexts, i.e., our short neutral stories, particularly for older adults could be attributed to higher inferential demands given that less contextual cues are available. Importantly though, this effect between reading time differences in the short versus long stories is nearly inexistent in the satisfaction condition. Further research needs to investigate if this effect is simply an artifact of our experimental design. If this is not the case, it is also worthwhile to understand better why older adults seem to benefit more from more contextual information in the case of change-of-state verbs.

Given the potential bias of our reading time paradigm, we now turn to the discussion of our offline data, for which the experimental bias does not hold and that are therefore more reliable.

### Offline processing of presuppositions (verification task)

Interesting patterns of results emerged from the offline verification task. First, there were no significant effects in participants’ accuracy to respond to the verification sentences. Second, a range of age-related effects emerged in the response times as depending on contextual availability, type of trigger, and mental workload. Both groups of participants were at ceiling in correctly answering to the target verification questions and no significant effects relative to mental workload, context and trigger emerged. Therefore, our participants recovered the presupposition triggered by both CSVs and DDs, independently of context (i.e., NEU vs. SAT) and mental load (i.e., Low vs. High). Most important, there were no group-related differences. Overall, this null effect is interesting because it suggests that the ability to update the discourse mental model with presupposed information is not compromised in late adulthood. This fully replicates Domaneschi and Di Paola (2019) and corroborates the idea that older adults do recover background information.

Results on the response times to verification sentences suggest instead that what seems to be compromised across the life span is the cognitive effort required to recover the presupposed information for updating with this the discourse mental model. The main patterns of results may be summarized as follows:

(i) *A range of significant main effects*. Response times were longer for older than younger adults (age-group effect); these were longer in the neutral context (context effect), in the condition of higher mental workload (mental load effect) and for change-of-state verbs than definite descriptions (trigger type effect).
(ii) *A range of significant interactions*, namely Group X Context and Group X Trigger.

As for (i), as expected, the main effect of age group reflects the general decline in information processing across the life span (e.g., Stine and Hindman 1994). The other main effects reveal patterns overall associated with the manipulations of the study. First, the longer response times in the condition of higher mental workload suggest that participants from both groups were burdened when asked to keep more information in mind. Therefore, as desired, manipulating the mental workload successfully taxed participants’ cognitive resources. Second, the longer response times for both groups of participants in the neutral context (vs. a supporting context) suggest that recovering presuppositions that were previously introduced in the context via accommodation requires a higher expenditure of mental resources. This is in line with previous studies showing that presupposition accommodation is costlier indeed than satisfaction (Schwarz 2007; Tiemann et al. 2015; Domaneschi and Di Paola 2018). Third, both groups of participants were slower at recovering a presupposition triggered by change-of-state verbs than that of definite descriptions. This result indicates higher recovering efforts associated with change-of-state verbs in general, and it is consistent with Domaneschi et al. (2014) who found extra difficulties precisely for this type of trigger. Following Domaneschi et al. we interpret this result as likely reflecting the higher cognitive demands associated with the complexity of the mental representation of change-of-state verbs. In fact, contrary to definite descriptions, change-of-state verbs require the representation of temporally displaced events that include both the event at a previous time and the event at the time of the utterance (i.e., X smoked and X has given up smoking).

More interesting for the purpose of our work are the significant interactions between Group and Context and between Group and Trigger, as well as the interaction between Load and Trigger that approached significance (*p* = 0.067) and is therefore worth a mention. The interaction between Group and Context revealed that, as compared to younger adults, older adults took longer at responding to the verification sentences of presuppositions that were presented within a neutral context. This suggests higher cognitive efforts in late adulthood associated with the recovery of a previously accommodated presupposition. In other words, recovering an accommodated presupposition becomes harder with normal aging. Considering that accommodation per se constitutes a more demanding condition in general, it is not surprising that this is even more so when the cognitive resources decline with aging. Overall, this result fits well with previous studies on presupposition accommodation (e.g., Domaneschi and Di Paola 2018; Schwarz 2015) and further enriches the picture of presuppositional skills across the life span adding the information that contextual availability makes a difference for presupposition recovery in old age: whether or not a presupposition is satisfied by the context does smooth, or increase, the cognitive effort underlying recovery.

As compared to younger adults, older adults recovered the presupposition of change-of-state verbs more slowly than that of definite descriptions (i.e., significant interaction between Group and Trigger). Therefore, it seems that the presuppositions of change-of-state verbs are more difficult to retrieve from memory. This pattern is corroborated by the interaction between Load and Trigger, which approached significance likely because of a small number of observations. Nonetheless, this interaction revealed a trend that is consistent with the present discussion: contrary to the trend for definite descriptions, participants’ response times to presuppositions activated by change-of-state verbs tended to be longer when their cognitive resources were more highly burdened. Taken together, these data suggest a peculiar difficulty relative to the recovery of change-of-state verbs rather than definite descriptions, indicating that change-of-state verbs seem more cognitively demanding—at least, when participants are asked to recover the correspondent presupposition within a task that overcharges their cognitive resources. The pattern fits smoothly with Domaneschi et al. (2014). In this work, the authors employed a dual task to assess participants’ recovery of change-of-state verbs (and other triggers) while taxing their mental load. Consistently with our findings, their results revealed that participants exhibited more difficulties in recovering change-of-state verbs (vs. definite descriptions and other triggers such as focus-sensitive particles) in the condition of high interference. Again, we follow Domaneschi et al. (2014) and interpret the extra difficulties for change-of-state verbs as mirroring higher costs associated with the complexity of a temporally displaced mental representation. In fact, this might explain the pattern of decay for change-of-state verbs: mentally representing change-of-state verbs might come at a surplus of cognitive demands that likely further slows recovering in late adulthood.

Beyond this, our results on change-of-state verbs are compatible with ERPs research showing that the higher costs for change-of-state verbs are linked to updating the mental model with their presuppositions, as mirrored by a greater P600 (Domaneschi et al. 2018).

Overall, the patterns of results revealed by this study are only partially in line with Domaneschi and Di Paola (2019). We found that the recovery of change-of-state verbs-related presuppositions was slower in the condition of higher mental workload, which suggests a role of working memory while recovering the presuppositions triggered by change-of-state verbs. This fits well with Domaneschi and Di Paola, who found that if on the one hand working memory did not ease recovering the presuppositions of definite descriptions, on the other hand, it played a role with change-of-state verbs. Together with Domaneschi et al. (2014), this set of studies argues in favor of a genuine involvement of working memory in presupposition processing and this issue would deserve even further investigation in future studies.

However, this study revealed older adults’ greater difficulties associated with change-of-state verbs. This does not replicate Domaneschi and Di Paola’s (2019) finding that older adults exhibited higher difficulties with the recovery of the presupposition of definite descriptions than that of change-of-state verbs. We explain the dissociation between studies in terms of differences in the materials as well as in task demands. Similar to Domaneschi and Di Paola (2019), we used both definite descriptions and change-of-state verbs. However, while Domaneschi and Di Paola used genitive constructions (e.g., *the pianist of the pub*), here we used simpler referential expressions (e.g., *the painting*). This difference might contribute explaining why our older adults exhibited no peculiar difficulty with definite descriptions. Most important, Domaneschi and Di Paola used only the change-of-state verb *to stop*. Conversely, here we used two change-of-state verbs, namely *to stop* and *to begin*. As we will discuss soon, the statistics on the response times for only change-of-state verbs revealed that older adults’ increased difficulty with the recovery of the presuppositions of change-of-state verbs was mostly driven by *begin* rather than *stop*. It might be the case that this effect obscured older adults’ difficulty associated with definite descriptions, thus contributing to explain the different patterns between studies. One more possible explanation for the divergence might be related to differences in task demands. Contrary to Domaneschi and Di Paola (2019), here we were particularly interested at the direct role of working memory and the cognitive load of presupposition processing. Therefore, we manipulated the mental workload and this manipulation likely increased the cognitive demands of the task in general.

To better explore older adults’ difficulties associated with change-of-state verbs, we analyzed participants’ accuracy and response times to the verification sentences that verified the presupposition of change-of-state verbs only, i.e., the comparison between *begin* vs *stop*. This analysis revealed no significant effects on participants’ accuracy, therefore confirming the idea that both groups updated the discourse mental model with presupposed information and that this ability is preserved with normal aging. Interestingly, instead, age-group differences emerged relative to the type of change-of-state verb on the response times. In particular, the interactions between Group and Verb Type and between Group, Load, and Verb Type were significant. These interactions revealed that (i) as compared to younger adults, older adults were slower recovering the presupposition triggered by *begin* than that of *stop*; and (ii) this was even more so in the condition of higher mental workload. Taken together, these data suggest that older participants exhibited higher cognitive efforts when dealing with *begin* than with *stop*. This further indicates that older adults’ difficulty with change-of-state verbs as emerged by the previous analyses might be driven by the fact that *begin* is somewhat more taxing for an older adult.

Why recovering the presupposition of *begin* seems more cognitively demanding than that of *stop*? We propose two interpretations. First, the target sentences containing *begin* were longer than those containing *stop*. Therefore, recovering the presupposition activated by a given trigger might turn harder when processing more linguistic material. A second, less simple, explanation might have to do with the nature of the mental representations involved in one case and the other. Both *begin* and *stop* involve the representation of temporally displaced events. However, *stop* involves the representation of an event, that already took place in the past and that has stopped in the present. Conversely, the underlying mental representation of *begin* includes an event that did not take place in the past, still has to take place and, for this reason, this may be more effortful. To better understand the difference regarding the recovery of *stop* vs *begin*, further research should investigate if the observed difference in the present study can be explained by the underlying aspectual differences between these verbs.

## Conclusion

To conclude, this study confirms that the ability to recover presupposed information declines with normal aging and reveals that the decline seems mostly linked to the cognitive load of the trigger and to the extent to which this hinges on working memory. Recovering the presupposition of change-of-state verbs is more cognitively demanding and requires higher working memory resources than definite descriptions, as suggested by the fact that both groups of participants tended to be slower at recovering the presupposition of change-of-state verbs in the higher mental workload condition. Importantly, this seems even more so in late adulthood presumably because the complexity of the temporally displaced mental representation increases the associated cognitive efforts.

## Figures and Tables

**Table 4:**
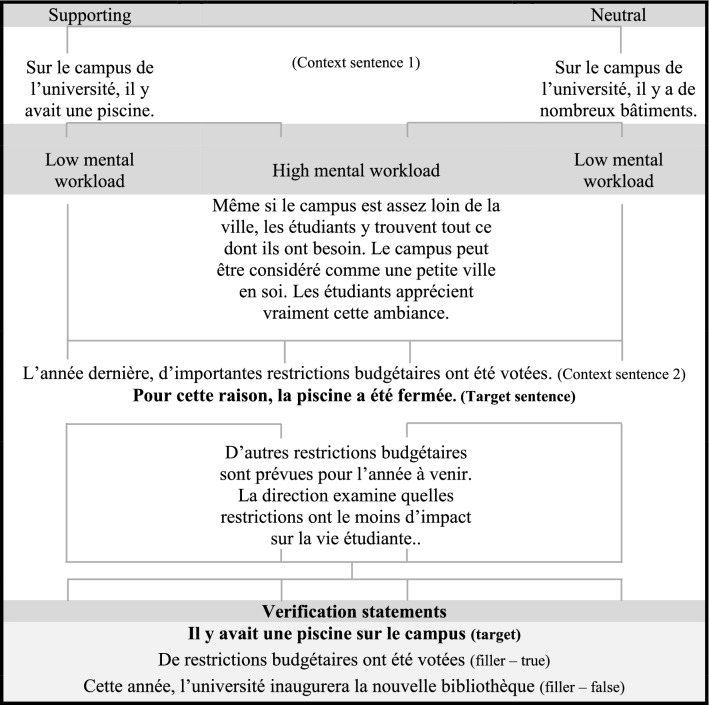
French Sample story for definite descriptions in the supporting and neutral condition

**Table 5:**
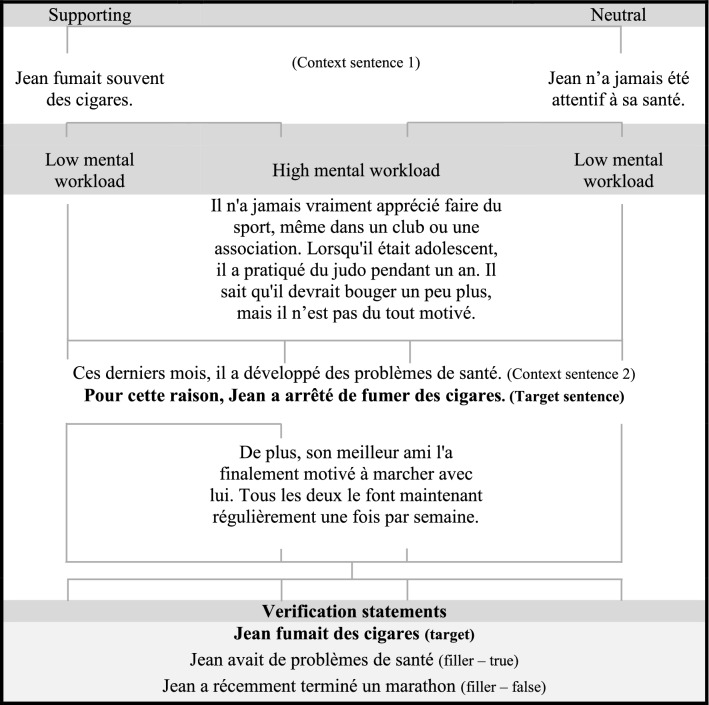
French Sample story for change of state of verbs in the supporting and neutral condition.

## Appendix

See Table 4, 5, 6, 7 and 8.

**Table 6:** Analyses of Variance results obtained with the lmerTest package from the LMMs analyses on the reading times at each region of the target sentence (i.e., Segment 1, Segment 2, Segment 3, and Total reading times (TOT))

	Target sentence region			
	Segment 1	Segment 2	Segment 3	TOT
Age group	*F*(1, 45.9) = 13.52; *p* < 0.0001	*F*(1, 45.7) = 39.73; *p* < 0.0001	*F*(1, 45.7) = 24.28; *p* < 0.0001	*F*(1, 45.8) = 24.81; *p* < 0.0001
Context	*F*(1, 239.1) = 4.02; *p* = 0.04	*F*(1, 259) = 5.92; *p* = 0.015	*F*(1, 228.4) = 10.73; *p* = 0.001	*F*(1, 258.4) = 8.95; *p* = 0.003
Mental workload	*F*(1, 308) = 10.08; *p* = 0.001	*F*(1, 308.6) = 5.14; *p* = 0.024	*F*(1, 311.4) = 0.33; *p* = 0.56	*F*(1, 309) = 4.36; *p* = 0.038
Trigger type	*F*(1, 307.9) = .12; *p* = 0.71	*F*(1, 308.5) = 67.39; *p* < 0.0001	*F*(1, 311.4) = 30.61; *p* < 0.0001	*F*(1, 308.9) = 24.29; *p* < 0.0001
Group X context	*F*(1, 129.7) = 0.73; *p* = 0.39	*F*(1, 136.1) = 1.79; *p* = 0.18	*F*(1, 64.1) = 2.94; *p* = 0.091	*F*(1, 66.5) = 0.95; *p* = 0.33
Group X load	*F*(1, 3396.6) = 0.01; *p* = 0.90	*F*(1, 3413.6) = 0.51; *p* = 0.47	*F*(1, 3318.4) = 0.28; *p* = 0.59	*F*(1, 3443.8) = 0.32; *p* = 0.57
Group X trigger	*F*(1, 3396) = 0.58; *p* = 0.44	*F*(1, 3413) = 22.21; *p* < 0.0001	*F*(1, 3317.8) = 2.25; *p* = 0.13	*F*(1, 3443.2) = 8.59; *p* = 0.003
Context X load	*F*(1, 307.9) = 4.92; *p* = 0.027	*F*(1, 308.6) = 6.29; *p* = 0.012	*F*(1, 311.4) = 6.48; *p* = 0.011	*F*(1, 308.9) = 7.64; *p* = 0.006
Context X trigger	*F*(1, 308) = 0.06; *p* = 0.79	*F*(1, 308.6) = 0.49; *p* = 0.48	*F*(1, 311.4) = 0.80; *p* = 0.36	*F*(1, 308.9) = 0.02; *p* = 0.88
Load X trigger	*F*(1, 307.9) = 1.01; *p* = 0.31	*F*(1, 308.5) = 0.33; *p* = 0.56	*F*(1, 311.4) = 0.38; *p* = 0.53	*F*(1, 308.9) = 0.67; *p* = 0.41
Group X context X load	*F*(1, 3395.9) = 2.12; *p* = 0.14	*F*(1, 3412.9) = 1.79; *p* = 0.18	*F*(1, 3317.7) = 3.06; *p* = 0.08	*F*(1, 3443.1) = 4.64; *p* = 0.031
Group X context X trigger	*F*(1, 3396) = 0.07; *p* = 0.78	*F*(1, 3413) = 0.19; *p* = 0.66	*F*(1, 3317.8) = 1.14; *p* = 0.28	*F*(1, 3443.2) = 0.07; *p* = 0.79
Group X load X trigger	*F*(1, 3395.9) = 5.01; *p* = 0.025	*F*(1, 3412.9) = 0.01; *p* = 0.91	*F*(1, 3317.7) = 0.35; *p* = 0.55	*F*(1, 3443.1) = 2.12; *p* = 0.14
Context X load X trigger	*F*(1, 308) = 0.13; *p* = 0.71	*F*(1, 308.6) = 0.40; *p* = 0.52	*F*(1, 311.4) = 0.17; *p* = 0.67	*F*(1, 309.0) = 0.25; *p* = 0.61
Group X context X load X trigger	*F*(1, 3396.6) = 3.01; *p* = 0.08	*F*(1, 3413.7) = 1.63; *p* = 0.20	*F*(1, 3318.5) = 0.0003; *p* = 0.98	*F*(1, 3443.8) = 1.68; *p* = 0.19

**Table 7:** Results on accuracy and response times to verification statements

	Accuracy, GLM	Response times, LMMs
Age group	*b* = − 0.015; SE = 0.35; *z* = − 0.045; *p* = 0.96	*F*(1, 43.81) = 31.59; *p* < 0.0001
Context	*b* = 0.134; SE = 0.36; *z* = 0.366; *p* = 0.71	*F*(1, 166.81) = 14.68; *p* = 0.0001
Mental workload	*b* = − 0.085; SE = 0.35; *z* = − 0.244; *p* = 0.81	*F*(1, 308.95) = 3.77; *p* = 0.053
Trigger type	*b* = − 0.23; SE = 0.34; *z* = − 0.676; *p* = 0.50	*F*(1, 308.92) = 45.39; *p* < 0.0001
Group X context	*b* = 0.488; SE = 0.54; *z* = 0.893; *p* = 0.37	*F*(1, 43.30) = 5.21; *p* = 0.027
Group X load	*b* = 0.406; SE = 0.51; *z* = 0.789; *p* = 0.43	*F*(1, 2310.09) = 0.25; *p* = 0.61
Group X trigger	*b* = − 0.297; SE = 0.46; *z* = − 0.641; *p* = 0.52	*F*(1, 2309.67) = 8.40; *p* = 0.004
Context X Load	*b* = 0.646; SE = 0.55; *z* = 1.160; *p* = 0.25	*F*(1, 308.93) = 0.04; *p* = 0.83
Context X trigger	*b* = 0.89; SE = 0.56; *z* = 1.583; *p* = 0.11	*F*(1, 308.93) = 1.74; *p* = 0.19
Load X trigger	*b* = − 0.107; SE = 0.47; *z* = − 0.23; *p* = 0.82	*F*(1, 308.92) = 3.36; *p* = 0.068
Group X context X load	*b* = − 0.832; SE = 0.81; *z* = − 1.015; *p* = 0.31	*F*(1, 2309.50) = 0.10; *p* = 0.75
Group X context X Trigger	*b* = 0.578; SE = 0.88; *z* = 0.658; *p* = 0.51	*F*(1, 2309.37) = 2.03; *p* = 0.15
Group X load X trigger	*b* = 0.256; SE = 0.67; *z* = 0.378; *p* = 0.71	*F*(1, 2309.77) = 0.25; *p* = 0.61
Context X load X trigger	*b* = − 0.452; SE = 0.81; *z* = − 0.554; *p* = 0.58	*F*(1, 308.96) = 0.08; *p* = 0.78
Group X context X load X trigger	*b* = − 0.772; SE = 1.21; *z* = − 0.635; *p* = 0.53	*F*(1, 2309.8) = 0.97; *p* = 0.32

Accuracy: results of the generalized linear model statistics (GLM); Response Times: analyses of variance results obtained with the lmerTest package from the linear mixed models (LMMs) statistics

**Table 8:** Results on accuracy and response times to verification statements for change-of-state verbs only (i.e., verb type: to begin versus to stop)

	Accuracy, GLM	Response times, LMMs
Age group	*b* = − 0.081; SE = 0.50; *z* = − 0.161; *p* = 0.87	*F*(1, 44.59) = 33.01; *p* < 0.0001
Context	*b* = 1.026; SE = 0.69; *z* = 1.487; *p* = 0.13	*F*(1, 61.90) = 7.66; *p* = 0.007
Mental workload	*b* = − 0.000; SE = 0.51; *z* = 0.000; *p* = 1	*F*(1, 145.92) = 14.8; *p* = 0.0001
Verb type	*b* = − 0.127; SE = 0.50; *z* = − 0.252; *p* = 0.80	*F*(1, 145.89) = 296.74; *p* < 0.0001
Group X context	*b* = 1.240; SE = 1.26; *z* = 0.979; *p* = 0.32	*F*(1, 44.24) = 1.04; *p* = 0.31
Group X load	*b* = 0.000; SE = 0.71; *z* = 0.000; *p* = 1	*F*(1, 2421.73) = 0.45; *p* = 0.49
Group X verb type	*b* = 0.127; SE = 0.70; *z* = 0.181; *p* = 0.85	*F*(1, 2420.67) = 34.03; *p* < 0.0001
Context X load	*b* = 1.495; SE = 6.08; *z* = 0.025; *p* = 0.98	*F*(1, 145.95) = 0.35; *p* = 0.55
Context X verb type	*b* = − 1.343; SE = 0.83; *z* = − 1.616; *p* = 0.10	*F*(1, 145.92) = 4.32; *p* = 0.039
Load X verb type	*b* = − 0.163; SE = 0.70; *z* = − 0.232; *p* = 0.81	*F*(1, 145.89) = 6.65; *p* = 0.011
Group X context X load	*b* = − 1.495; SE = 6.08; *z* = − 0.025; *p* = 0.98	*F*(1, 2420.28) = 0.20; *p* = 0.65
Group X context X verb type	*b* = − 0.923; SE = 1.43; *z* = − 0.644; *p* = 0.52	*F*(1, 2419.12) = 1.05; *p* = 0.31
Group X load X verb type	*b* = 0.965; SE = 1.05; *z* = 0.913; *p* = 0.36	*F*(1, 2420.67) = 5.50; *p* = 0.019
Context X load X verb type	*b* = − 1.442; SE = 6.08; *z* = − 0.024; *p* = 0.98	*F*(1, 145.92) = 0.10; *p* = 0.75
Group X context X load X verb type	*b* = 1.38; SE = 6.08; *z* = 0.023; *p* = 0.98	*F*(1, 2419.12) = 0.02; *p* = 0.87

Accuracy: results of the generalized linear model statistics (GLM); Response Times: analyses of variance results obtained with the lmerTest package from the linear mixed models (LMMs) statistics

## Footnotes

1 In this work, we are treating presuppositions along a tradition of linguistic research according to which a new information can be introduced in the context by an utterance that either *asserts* or *presupposes* that content—leading the hearers to *accommodate* it (Karttunen 1974; Stalnaker 1974; Heim 1992). Note, however, that some authors have contested the idea that accommodation is real and that presuppositions are accommodated, by proposing that presuppositions are assimilated into the asserted content (e.g., Gazdar 1979; van der Sandt 1992, Gauker 1998). This perspective does not predict differences in terms of conversational appropriateness between asserting implausible or controversial information and presupposing it—see Singh et al. (2016) for evidence against this prediction.

2 It is worth mentioning that the theoretical literature has proposed up to now two main ways of modelling the semantics of presuppositions. On the one hand, according to Heim’s Context Change Semantics (CCS) (Heim 1983, 1992; Kratzer and Heim 1998), presuppositions are conventional properties of lexical items, and constitute a precondition for the utterance comprehension. On the other hand, among the lexical approaches to presuppositions, the Discourse Representation Theory (DRT—Kamp 1984; Kamp and Reyle 1993) proposes an alternative view to Heim’s. According to DRT, presupposition triggers are anaphoric expressions and, similarly to pronouns, activate a process of anaphoric retrieval of a proper antecedent in the previous discourse structure. One of the main consequences of this view is that all trigger types are considered anaphoric expressions that prompt an anaphoric procedure of antecedent retrieval. Beside these differences, for the purpose of our paper, it is important to underline that, equally to Heim’s view, DRT also considers presuppositions as lexical properties of the presupposition triggers: in this view, if a suitable referent for the presupposition introduced by a trigger is not available in the preceding discourse structure, a new one—to which the presupposition is linked—is accommodated. Even though for the sake of our paper we decide to treat presuppositions and presupposition triggers within a dynamic approach à la Heim, we think it is important to stress that modelling presupposition triggers at a theoretical level as anaphoric expressions would not affect significantly the main point of our work which is about how presuppositional skills vary across the life span.

3 In psycholinguistics, online measures are aimed at tapping online processing, that is the processes underlying a given linguistic phenomenon while the given linguistic item (e.g., a word, a segment, a sentence) unfolds (e.g., Vorwerg 2012). Common experimental techniques used to collect online measures are reading time paradigms, eye-tracking paradigms as well as neurophysiological techniques such as EEG and fRMI. In contrast, offline measures provide insights into language comprehension after real-time language processing takes place, e.g., accuracy scores, acceptability judgments, etc. In the present study, we are interested in how presuppositions are processed in real time, which are assessed via the means of the reading time paradigm. In addition, we are interested in the update of the mental model with the presupposed content, which is measured by means of accuracy as well as response times to verification statements related to the content of the sentence.

4 It is important to note though that the difference between satisfied and accommodated definite descriptions also depends on the degree of inferential demand. For instance, O’Brien et al. (1988) results indicate that the difference between satisfied and accommodated definite descriptions only exists in loosely constraining contexts, but does not emerge in highly constraining contexts (see also Burkhardt 2007, who compared necessary vs probable vs inducible contexts using an ERP paradigm—depending on the inferential demand distinct ERPs emerged in the latter two conditions when compared to the necessary one). Similar findings are also found with respect to anaphor processing; the degree of explicitness (explicit versus implicit referents) has a much larger effect in the condition in which the referent is not central to the argument (e.g., Cornish et al. 2005).

5 Even though it is consistently reported in the literature that there is a difference between neutral vs. supporting contexts, it is important to mention that there is neither a difference in acceptability nor in reading time between asserted—an indefinite—and presupposed content—a definite—in plausible contexts (Singh et al. 2016). However, the results by Xia et al. (2019) using an EEG paradigm show that introducing indefinites and definites in plausible contexts involve different processes, i.e., definites trigger a LAN (for an interesting proposal of the underlying mechanisms, please see Xia et al. 2019).

6 For the other presupposition triggers, the accuracy scores did not differ between the low vs high interference condition (low interference condition: factive verbs (86%), definite descriptions (86%), focus-sensitive particles (58%); high interference condition: factive verbs (90%), definite descriptions (89%), focus-sensitive particles (60%).

7 In Domaneschi and Di Paola (2019), the used reading time paradigm for the target sentence was a word-by-word paradigm, whereas in our material, we used a segment-by-segment reading time, which already started at the second context sentence. This paradigm choice appeared to be more adapted for longer scenarios as this was the case in our experiment (see, e.g., Fossard et al. 2012, who also used a segment-by-segment paradigm in longer scenarios).

8 In Table 1, we only present the high cognitive workload condition. The low cognitive workload condition is similar, the only difference being that the two distractor sentence blocks are not included (for a more detailed presentation of the low cognitive workload condition, see Table 2 of Method section).

9 All stimuli as well as all other materials relevant for the later described pretests as well as the results can be accessed here: https://osf.io/4vahf/?view_only=53a5c22912344ebdb5a5fd5e02b94ccb. To access all stimuli, please access the following folder: *3_Experimental stimuli.*

10 After the analysis of the second pretest, the results revealed that three pairs for each trigger type showed a significant difference. Given that the overall results show that the two versions for each trigger type did not differ significantly, we terminated our norming study here.

11 That is, one that included random intercept for subjects and items as well as by-subjects and by-items random slopes for Context, Mental Workload, and Trigger Type.

12 A generalized linear-mixed model statistics (GLMM) was first conducted. However, because the random effects variance for this model was very close to zero, the GLMM did not converge. Beyond this, the absence of random variance made the GLMM statistics unjustified for the accuracy data. Therefore, a generalized linear model statistics was conducted.

13 As before, accuracy was analyzed with generalized linear model statistics; response times were analyzed with LMMs statistics. The random effects structure of the LMM model included random intercept for subject and items and by-subject random slope for Context. The fixed structure included Age Group, Context, Mental Workload, Verb Type (i.e., arrêter vs. commencer), and the resulting interactions.
